# Myofibroblast transdifferentiation of keratocytes results in slower migration and lower sensitivity to mesoscale curvatures

**DOI:** 10.3389/fcell.2022.930373

**Published:** 2022-07-22

**Authors:** Cas van der Putten, Daniëlle van den Broek, Nicholas A. Kurniawan

**Affiliations:** ^1^ Department of Biomedical Engineering, Eindhoven University of Technology, Eindhoven, Netherlands; ^2^ Institute for Complex Molecular Systems, Eindhoven University of Technology, Eindhoven, Netherlands

**Keywords:** cell migration, substrate curvature, corneal keratocyte, myofibroblast transdifferentiation, stress fibers

## Abstract

Functional tissue repair after injury or disease is governed by the regenerative or fibrotic response by cells within the tissue. In the case of corneal damage, keratocytes are a key cell type that determine the outcome of the remodeling response by either adapting to a fibroblast or myofibroblast phenotype. Although a growing body of literature indicates that geometrical cues in the environment can influence Myo(fibroblast) phenotype, there is a lack of knowledge on whether and how differentiated keratocyte phenotype is affected by the curved tissue geometry in the cornea. To address this gap, in this study we characterized the phenotype of fibroblastic and transforming growth factor β (TGFβ)-induced myofibroblastic keratocytes and studied their migration behavior on curved culture substrates with varying curvatures. Immunofluorescence staining and quantification of cell morphological parameters showed that, generally, fibroblastic keratocytes were more likely to elongate, whereas myofibroblastic keratocytes expressed more pronounced α smooth muscle actin (α-SMA) and actin stress fibers as well as more mature focal adhesions. Interestingly, keratocyte adhesion on convex structures was weak and unstable, whereas they adhered normally on flat and concave structures. On concave cylinders, fibroblastic keratocytes migrated faster and with higher persistence along the longitudinal direction compared to myofibroblastic keratocytes. Moreover, this behavior became more pronounced on smaller cylinders (i.e., higher curvatures). Taken together, both keratocyte phenotypes can sense and respond to the sign and magnitude of substrate curvatures, however, myofibroblastic keratocytes exhibit weaker curvature sensing and slower migration on curved substrates compared to fibroblastic keratocytes. These findings provide fundamental insights into keratocyte phenotype after injury, but also exemplify the potential of tuning the physical cell environments in tissue engineering settings to steer towards a favorable regeneration response.

## Introduction

**FIGURE 1:**
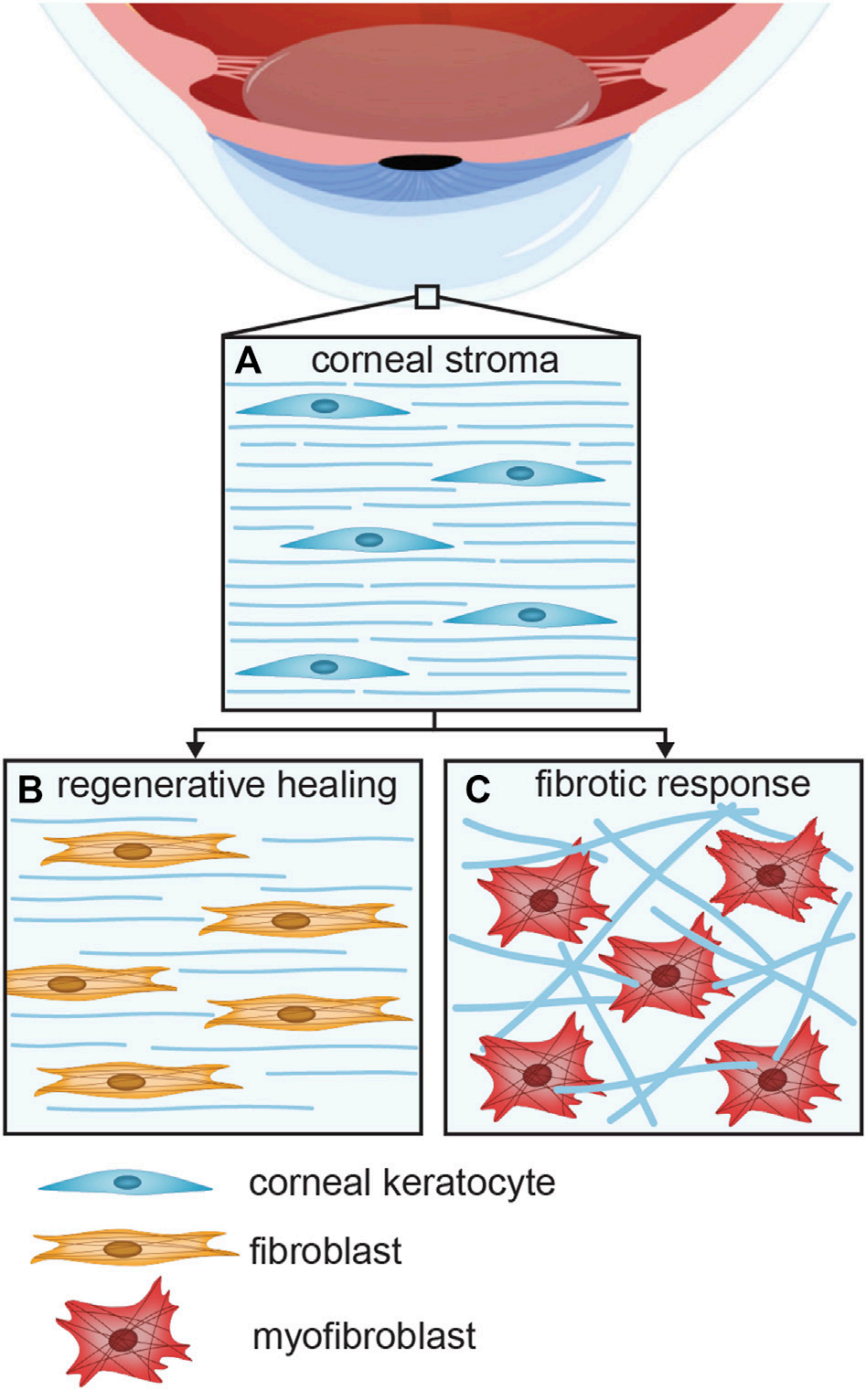
The various keratocyte phenotypes present in the **(A)** healthy, **(B)** regenerative, and **(C)** fibrotic corneal stroma. Activation of normally quiescent keratocytes leads to the differentiation of fibroblasts that proliferate and migrate towards the damaged area, where the ECM is regenerated. Hyperactivation of keratocytes may lead to a fibrotic response.

In the event of an injury to the cornea, the cellular wound healing response can be regenerative, leading to restoration of the corneal tissue function, or fibrotic, resulting in impaired vision or blindness (Jhanji, Billig and Yam, 2021). Keratocytes, the native cell type of the corneal stroma (Figure 1A), are a key cell type in both the regenerative (Figure 1B) and fibrotic responses (Figure 1C) (West-Mays and Dwivedi, 2006). Upon injury of the corneal stroma, the wounded area is populated by myofibroblasts, derived from either bone-marrow derived precursor cells or activated keratocytes (Medeiros et al., 2018; Yam et al., 2020). In the latter, normally quiescent keratocytes are activated into repair phenotypes, induced by cytokines released from the epithelial layer as the epithelial basement membrane and Bowman’s layer are disrupted (Yam et al., 2020). The activation of keratocytes leads to differentiation into fibroblasts that start to proliferate, migrate to the damaged site, and produce extracellular matrix (ECM) proteins to regenerate healthy tissue (Menko, Walker and Stepp, 2020). However, these fibroblasts may be hyperactivated into myofibroblasts that give the wound healing response a fibrotic character. A key signaling molecule that induces the transition from fibroblast to myofibroblast is transforming growth factor β (TGFβ). As a result of fibrosis, corneal transparency is decreased, as myofibroblasts deposit ECM proteins that form disorganized, opaque scar tissue, with altered composition, in strong contrast with the highly regular arrangement of thin collagen fibrils in the healthy corneal stroma (Jester et al., 1999; Medeiros et al., 2018). Furthermore, myofibroblasts cause tissue contraction that might alter the specific refractive capacity of the cornea (Stramer et al., 2003; Fini and Stramer, 2005). Myofibroblast contraction induces a mechanical feedback loop that leads to progression of fibrosis: the cells modify the structure of the ECM, and the altered ECM structure contributes to the myofibroblast phenotype and behavior (D’Urso and Kurniawan, 2020). Ultimately, fibrosis may result in hazy vision or even blindness (Flaxman et al., 2017).

Corneal keratocytes have been shown to display distinct migration and contractile behaviors in the different stages of cell activation during the fibrotic wound-healing response. Transdifferentiation of keratocytes into myofibroblasts by serum growth factors, including TGFβ, was shown to induce cell proliferation and contraction (Jester and Ho-Chang, 2003). Besides, fibroblastic features of keratocytes when cultured in high-glucose condition were associated with more pronounced cell migration and contraction (Foster, Gouveia and Connon, 2015). The enhanced migratory behavior of activated keratocytes with a fibroblastic phenotype compared to the quiescent state has also been shown in three-dimensional (3D) compressed collagen matrices (Kim et al., 2010). On the other hand, myofibroblastic keratocytes migrated less than fibroblastic cells, but caused more contraction and reorganization of the ECM (Kim et al., 2010). Cell migration behavior and force generation appear to be dependent in a dose-dependent manner on the cellular pathways that are stimulated upon exposure to specific cytokines. Particularly important is the Rho-GTPase/Rho-kinase pathway, which leads to the assembly of actin stress fibers (Machesky and Hall, 1997) and myosin II-dependent cell contractility (Levayer and Lecuit, 2012). This pathway is activated by TGFβ, but also by factors present in fetal bovine serum (FBS) in the cell culture medium, such as lipophosphatidic acid (Petroll et al., 2008). These findings suggest that the altered cellular migration behavior of activated keratocytes may play an important role during fibrosis.

Recent works have shown that the geometry of tissues and cellular substrates can strongly influence a variety of cellular characteristics, including morphology, adhesion, and migration (Werner, Kurniawan and Bouten, 2020), all of which are vital processes during the activation of keratocytes as well as the wound healing process and the fibrotic progression in the corneal stroma. Meso- and macro-scale curvatures are a common geometrical feature in many tissues (Assoian et al., 2019; Baptista et al., 2019; Callens et al., 2020), and is critical for the optical function of the cornea to refract light. When cultured on substrates with convex and concave regions, several cell types, such as mesenchymal stem cells and fibroblasts, were shown to position themselves predominantly in concave curvatures (Pieuchot et al., 2018), indicating that cells can sense and respond to non-planar substrate curvature. When the curvature is anisotropic (e.g., cylinders), mesenchymal stromal cells were shown to align and migrate along the longitudinal axis of convex cylinders, but stretch over concave cylinders without a preferential orientation (Werner et al., 2019). The sensing of curvature by cells has also been shown in the context of collective cell behavior: NIH3T3 fibroblasts in a confluent monolayer also aligned along the longitudinal axis of concave cylinders (Yip, Huang and Chiam, 2018). Similar behavior was observed for dense monolayers of osteoblast-like MC3T3-E1 cells on concave and convex cylinders, as both collective cell alignment and polarization were affected by the curved environment (Liu et al., 2018). The directional response of cells to substrate curvature seems to be cell-type-dependent, as Madin Darby Canine Kidney (MDCK) and renal epithelial cells as well as endothelial cells tend instead to wrap around convex cylinders and align in the circumferential direction (Fioretta et al., 2014; Yevick et al., 2015). Interestingly, mesoscale curvatures (up to length scales of millimeter) can even overrule competing effects from nano- and micro-scale contact and topographical cues (Werner et al., 2018; van der Putten et al., 2021). Furthermore, template substrates with macroscale curvatures (in order of millimeters) have also been shown to induce alignment of layers of corneal keratocytes and their produced ECM (Gouveia et al., 2017). Despite this growing body of evidence, how the differentiated phenotypes of corneal keratocytes are influenced by tissue curvature during their activation is still poorly understood.

In this study, we investigated the impact of TGFβ-induced transdifferentiation of fibroblasts into myofibroblasts on the curvature-dependent adhesion, morphology, and migration of human corneal keratocytes. To study this systematically, we used a 2.5D *in vitro* experimental platform, employing a microfabricated chip made from polydimethylsiloxane (PDMS), containing well-defined curved cylindrical structures with sizes ranging from 125 μm, i.e., comparable to cell size, to 2000 μm, which more resembles physiological tissue curvatures. This approach allowed us to quantitatively decouple the effects of TGFβ-induced transdifferentiation and substrate curvature on the keratocyte migration modes evoked by these cues. The findings thus reveal a previously unknown phenotype-dependent response to curvature that may play a role during corneal wound healing and regeneration.

## Materials and methods

### Human corneal keratocyte cell culture

Human corneal keratocyte (HCK) cell lines were obtained as a kind gift from Dr. Zorn-Kruppa (University Medical Center Hamburg). The HCK cell line was derived from the human corneal stroma and immortalized through SV-40 transfection, and has been previously demonstrated to mimic both the phenotype and the response to growth factor stimulations of their primary precursors (Zorn-Kruppa et al., 2005). HCK cells were cultured in Dulbecco’s Modified Eagle’s Medium (DMEM, Sigma-Aldrich) supplemented with 5% FBS (Biochrom) and 1% penicillin/streptomycin at 37°C and 5% CO_2_ in T25 flasks until 80% confluency was reached. Due to activation of the cells by the FBS supplementation, the HCK cells can be considered activated keratocytes, resembling a fibroblastic phenotype. As a comparison, quiescent keratocytes, hereafter referred to as HCK^−^, were cultured in keratinocyte growth medium (KGM), consisting of serum-free keratinocyte basal medium (KBM, Lonza), KGM Single Quots (Lonza), and 0.5 mM calcium chloride (Agros Organics) (Manzer et al., 2009). With Single Quots supplements, KGM contains 30 μg/ml bovine pituitary extract, 0.1 ng/ml human epidermal growth factor (hEGF), 0.5 μg/ml hydrocortisone, 5 μg/ml insulin, 15 ng/ml amphotericin B, and 30 μg/ml gentamicin sulfate. Cells were passaged twice a week, and cells between passages 5 and 10 were used for the experiments described in this study.

### Phenotypic characterization with immunofluorescence staining, imaging, and analysis

For phenotypic evaluation, all cells were cultured on cover glasses and analyzed using immunofluorescence staining. Cover glasses were sterilized using ethanol, washed 3 times with sterile PBS and coated using a neutralized (pH 7.4) 0.3 mg/ml bovine collagen I (PureCol, Advanced Biomatrix) overnight at 4°C. The next day, the excess collagen was removed and the cover glasses were washed with PBS. HCK cells, both with and without TGFβ supplementation were seeded at a density of 12,000 cells/ml. HCK cells that received TGFβ supplementation during culture, hereafter referred to as HCK + TGFβ cells, were allowed to adhere to the surface for 6 h before TGFβ1 (Peprotech) was added to a final concentration of 10 ng/ml. The cells were cultured for 4 days under these conditions to allow differentiation to the myofibroblast phenotype, following previously validated protocols (Zorn-Kruppa et al., 2005; Manzer et al., 2009; Engelke et al., 2013; Turan et al., 2021). HCK^−^ cells were seeded at a density 50,000 cells/ml and cultured for 1 day.

For immunofluorescence staining, samples were washed twice with PBS, fixed with 3.7% formaldehyde for 15 min, washed three times with PBS, permeabilized with 0.1% Triton-X100 in PBS for 10 min, and treated with 5% BSA in PBS for blocking. The samples were incubated overnight with primary monoclonal antibodies that bind to α-smooth muscle actin (α-SMA) [mouse-IgG2a anti- α-SMA (1:300 dilution, a2547, Sigma-Aldrich)] and vinculin [rabbit-IgG anti-vinculin (1:300, 42H89L44, Thermo Scientific)]. After washing, the following secondary antibodies were used to visualize primary antibodies: goat anti-mouse-IgG2a with Alexa Fluor 488 (1:250, A21131, Molecular Probes) and donkey anti-rabbit-IgG with Alexa Fluor 555 (1:250, A31572, Invitrogen). F-actin was visualized by staining with phalloidin-atto647N (Sigma-Aldrich), and nuclei were visualized with DAPI. The cover glasses were transferred to microscopy slides with Mowiol mounting medium.

The cells were imaged with a Leica TCS SP8X confocal microscope using HC PL APO CS2 objectives (20×/0.75, 40×/0.95, and 63×/1.40), and images were processed in ImageJ. Quantitative image analysis was performed with CellProfiler software (Carpenter et al., 2006). Three images (20× magnification) were analyzed for each experimental group (*n* > 215 cells per group). The nuclei (primary objects) and F-actin cytoskeleton (secondary objects) were identified, and the F-actin staining was used to define cell outlines. Next, the size and shape of the identified cells was analyzed. The following parameters were taken into account: length of the major cell axis, area, eccentricity, and solidity. Eccentricity is defined as the ratio of the distance between the foci of the ellipse fitting the cell and its major axis length, with the value ranging from 0 for a circle and 1 for a line. Solidity is defined as the proportion of the pixels in the convex hull that are also in the identified object and indicates how irregular the cell shape is.

### Curvature chips

**FIGURE 2:**
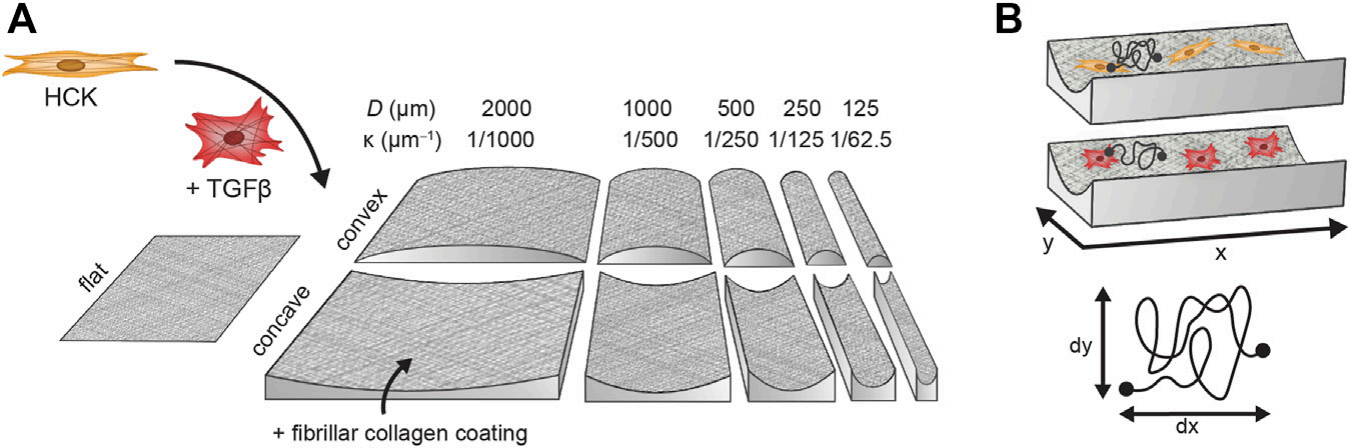
Experimental workflow of fibroblast (HCK) and myofibroblast (HCK + TGFβ) migration on flat substrates and semi-cylinders with diameters between 2000 µm ( κ = 1/1000 μm^−1^) and 125 µm ( κ = 1/62.5 µm^−1^). **(A)** Cell adhesion and morphology of both cell phenotypes are captured on convex and concave cylinders. **(B)** Migration behavior is captured over time and quantified on concave cylinders.

Polydimethylsiloxane (PDMS) cell culture chips containing concave and convex semi-cylindrical structures were produced using a molding process as previously designed and described (Werner et al., 2018). The curvature on any point on the chip surface can be characterized in terms of the principal curvature κ=2/D , where D is the diameter of the osculating circle at the point of interest on the curvature. Cylinders with diameters of D = 125, 250, 500, 1000, and 2000 µm were used in this study, corresponding to principal curvatures κ = 1/62.5, 1/125, 1/250, 1/500, and 1/1000 μm^−1^, respectively, in the circumferential direction. All cylinders have a depth or height of 170 µm and a length of 1000 µm (Figure 2). For brevity, the structures are hereinafter referred to by their diameters rather than principal curvatures. PDMS (Sylgard 184, with a 1:10 curing agent:elastomer ratio, Dow Corning) was poured on a glass mold (FEMTOPrint) produced and prepared as described previously (Werner et al., 2018) and cured overnight to produce a positive imprint of the glass mold. The imprint was subsequently used for the production of a PDMS mold that contains the negative imprint of the curvatures by pouring PDMS on the imprint and curing for 3 h at 65°C. Multiple cell culture chips were produced by pouring PDMS into the mold and curing for 3 h at 65°C. Tridecafluoro (1,1,2,2-tetrahydrooctyl)trichlorosilane (AB111444, ABCR) was applied too all PDMS imprints and mold to avoid the irreversible binding of PDMS layers. To allow live-cell imaging in 6-well glass-bottom plates, the chips were cut into a fitting size before protein coating.

### Cell migration experiments on curvature chips

To facilitate cell attachment, the PDMS chips were coated with fibrillar collagen (Figure 2) (Werner et al., 2018). First, the chips were disinfected with ethanol and washed three times with sterile PBS. Residual PBS droplets were removed and the chips were exposed to UV-ozone for 16 min to enhance binding between PDMS and collagen. The surface of the chips was then wetted with PBS to facilitate spreading of the collagen solution and 0.3 mg/ml neutralized (pH 7.4) bovine collagen I solution was distributed evenly over the chips. The fibrillar collagen coating was allowed to form overnight at 4°C. Afterwards, excess collagen was removed and the chips were washed with PBS. The collagen coating was confirmed by staining with collagen-binding protein CNA35 labelled with Oregon Green 488 (Aper et al., 2014).

For the HCK cells activated with TGFβ, the cells were incubated with 10 ng/ml TGFβ in a T25 flask for 4 days prior to seeding on the chips. Both HCK and HCK + TGFβ cells were seeded on the chips by incubating them with a seeding density of 120,000 cells/ml on the chips for 24 h. Cells adhering on the chips were stained with 5 µM CellTracker Orange in serum-free medium for 20 min, after which the culture medium was refreshed and the cells were incubated for another 6 h. Live-cell confocal imaging was done using a Leica TCS SP8X microscope equipped with a cage incubator system that allows imaging at 37°C and 5% CO_2_. The time-lapse imaging was performed using a 10×/0.40 objective. The collagen staining was used to define Z-stacks for cylinders and surrounding flat areas with a Z-spacing of 4 µm. The cells on these areas were imaged every 20 min for 22 h with a resolution of 512 × 512 pixels.

### Cell migration analysis

Maximum intensity projections were made for all imaged areas in ImageJ (Schindelin et al., 2012). Following our analysis in a previous study, cell movement in the Z-direction is negligible for D of 500 µm or above, thus the analysis of cell migration trajectory was performed directly using the maximum intensity projection images (Werner et al., 2019). For the smallest cylinders with D = 125 µm and D = 250 μm, the images were pre-processed to remap the cylindrical geometry into planar images using a custom MATLAB script. The centroids of the cells were tracked manually with the ImageJ plugin MTrackJ (Meijering, Dzyubachyk and Smal, 2012). At least 26 cells were tracked for each cylinder size and flat area per condition, with a total of 523 cell trajectories being analyzed. The directionality in the migration behavior is indicated as the directionality index dx/dy , where dx is maximum displacement along the longitudinal axis of the cylindrical structures, and dy the maximum displacement along the circumferential axis of the cylindrical structures. The migration speed was calculated from the displacement of each tracked cell after each time interval, and the average speed of each cell was taken. The mean-squared displacement (MSD) for each experimental group was calculated as the ensemble averaged squared displacement $\Delta r^{2}$ . A track segment was considered to be “aligned” when the orientation that the track segment makes with respect to the long axis of the cylindrical structure is less than 30°. “Alignment” on the flat areas of a chip was taken with respect to the long axis of cylindrical structures on the same chip. All trajectory analyses were performed in MATLAB (The Mathworks Inc.).

### Statistical analysis

Statistical analysis was performed to evaluate if there are significant differences in cell morphologies in the immunofluorescence images, and in migration parameters of cells in time-lapse imaging experiments. Non-parametric Kruskal-Wallis tests and Dunn’s multiple comparison tests with Benjamini-Hochberg *p*-adjustment method were executed in R studio or MATLAB. The numbers of analyzed cells and the resulting *p*-values are indicated in the figure captions.

## Results

### TGFβ treatment affects the phenotype, morphology, and adhesion of human corneal keratocytes

**FIGURE 3:**
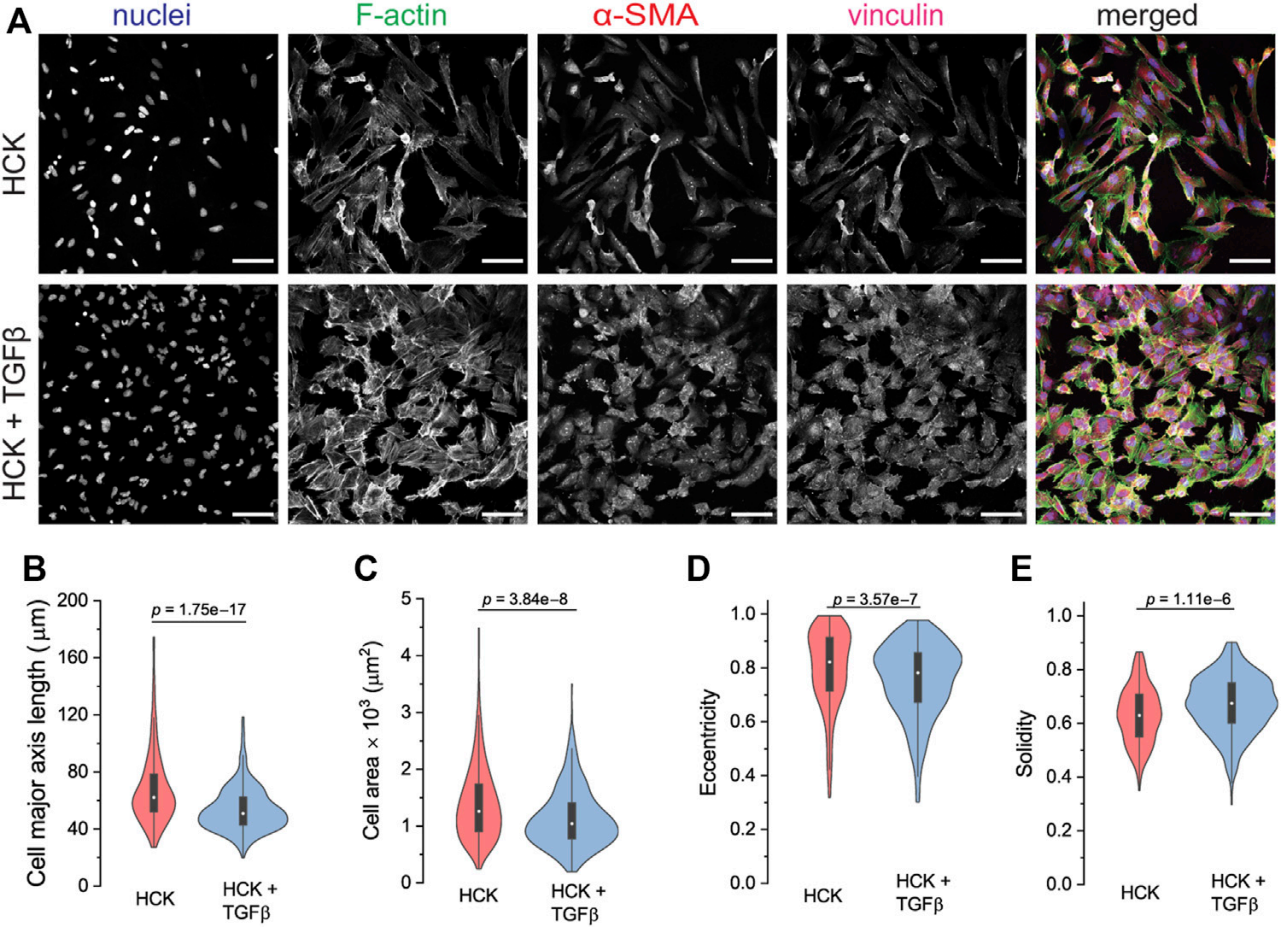
Cell phenotype and morphological analysis of HCK and HCK + TGFβ cells. **(A)** Immunofluorescence staining of HCK and HCK + TGFβ cells, showing the fibroblast and myofibroblast phenotypes, respectively. Cells were stained for nuclei (Dapi, blue), F-actin (phalloidin, green), α-SMA (red), and vinculin (magenta). Scale bar: 100 µm. **(B–E)** Cell morphological quantification of **(B)** major axis length, **(C)** cell area, **(D)** eccentricity, and **(E)** solidity of both HCK and HCK + TGFβ cells. *n* = 247 HCK cells and 512 HCK + TGFβ cells.

As a model of the cells involved in the wound healing and regeneration of the corneal stroma after injury, we used the human corneal keratocyte (HCK) cell line (Zorn-Kruppa et al., 2005). HCK cultured in the presence of 5% FBS resembled active fibroblastic keratocytes with an elongated morphology and actin stress fibers directed largely in the long axis of the cells (Figure 3A, top panel). The cells also expressed cytoplasmic α-SMA with no specific architecture and no colocalization with the actin stress fibers. As a comparison for quiescent keratocytes (Jester et al., 1994), HCK^−^ cells were less activated than HCK cells as they exhibited a more flattened morphology, less developed stress fibers, no visible focal adhesions, and the cells expressed negligible α-SMA (Supplementary Figure S1).

**FIGURE 4:**
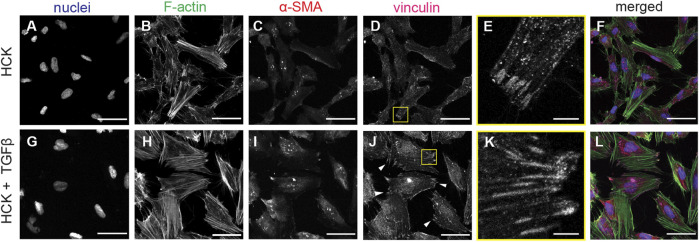
F-Actin and vinculin localization in HCK and HCK + TGFβ cells. **(A–F)** HCK cells and (**G**–**L**) HCK + TGFβ cells stained for **(A,G)** nuclei (Dapi, blue), **(B,H)** F-actin (phalloidin, green), **(C,I)** α-SMA (red), and **(D,J)** vinculin. Scale bars: 50 µm. **(E,K)** Zoom-in views of **(D)** and **(J)**, respectively, showing the formation of focal adhesions. Scale bar: 5 µm.

To induce transdifferentiation into myofibroblasts, we added TGFβ to the HCK culture. Under this condition, the HCK cells exhibited a less elongated morphology, more developed F-actin stress fibers, and more pronounced α-SMA and focal adhesions (Figure 3A, bottom panel). This is consistent with the expectation for myofibroblastic phenotype (Tomasek et al., 2002). Quantitative morphometric analysis of the two culture conditions indicated that HCK cells were longer (Figure 3B) and had larger spread area (Figure 3C) in the absence of TGFβ, compared to in its presence. These suggested that TGFβ treatment led to reduced cell elongation and less, shorter protrusions, as was indeed quantified through lower eccentricity (Figure 3D) and higher solidity (Figure 3E). Moreover, with TGFβ treatment, the cells form more mature focal adhesions, as indicated by the long vinculin structures at the end of the actin stress fibers, which are much less observed in the absence of TGFβ (Figure 4). Our observations indicate that HCK cells in the presence of 5% FBS were activated by TGFβ into the myofibroblast phenotype. These results demonstrate that the HCK cell line, coupled with TGFβ treatment, can be used as a simple cell model for investigating the impact of myofibroblastic transdifferentiation on corneal keratocyte’s environment sensing and migration.

### Corneal keratocytes adhered on concave but not convex substrates

We next investigated whether keratocyte adhesion and migration are affected by substrate curvature by seeding the cells on convex and concave cylindrical structures in our chips. To facilitate cell adhesion on the PDMS chips and to mimic the nanoscale architecture of the corneal stroma, the chips were coated with a thin layer of fibrillar collagen (Werner et al., 2018). Interestingly, while HCK cells adhered to the concave cylinders and the flat areas on the chip, we found that the cells generally had trouble maintaining stable adhesion to convex cylinders, both with and without TGFβ treatment. In particular, the cells stayed rounded and detached easily from the convex surface. This is in contrast to the behavior of human mesenchymal stromal cells, which readily adhered to convex surfaces of similar curvature (Werner et al., 2018), but is reminiscent of other cell types such as human endothelial cells, which also failed to adhere to convex substrates (van der Putten et al., 2021). Aside from this cell-type dependence, one possible reason for this observation is that adhering to convex substrates are energetically less favorable for contractile cells. Forces generated by the stress fibers can cause bending of the cell body and indentation of the nucleus (Anselme et al., 2018) (Werner et al., 2017), which can further affect cellular decision making (Li et al., 2014). Indeed, it has been widely reported that cells prefer to stay within, or even actively migrate towards, concave areas (Pieuchot et al., 2018), and they try to avoid curvature by reorienting in the direction of the minimal positive (convex) curvature (Werner et al., 2019).

After a prolonged incubation of 4 days, we observed some HCK cells adhering on the convex cylinders (Supplementary Figure S2). We suspect that cells on the concave and flat areas on the chips started to migrate onto the convex cylinders upon reaching a high enough confluency, similar to an earlier observation for renal epithelial cells (van Gaal et al., 2021) and keratocytes on convex domes (Gouveia et al., 2017). The cells on the convex cylinders tended to align strongly along the longitudinal axis of the cylinders, similar to the behavior of human mesenchymal stromal cells on convex cylinders. However, the number of cells was low and the cells exhibited an unhealthy morphology that is uncharacteristic of fibroblastic keratocytes. Thus, in the remainder of the study we decided to focus on the HCK cells with and without TGFβ on concave cylinders.

### Keratocyte transdifferentiation affects migration speed on concave substrates

**FIGURE 5:**
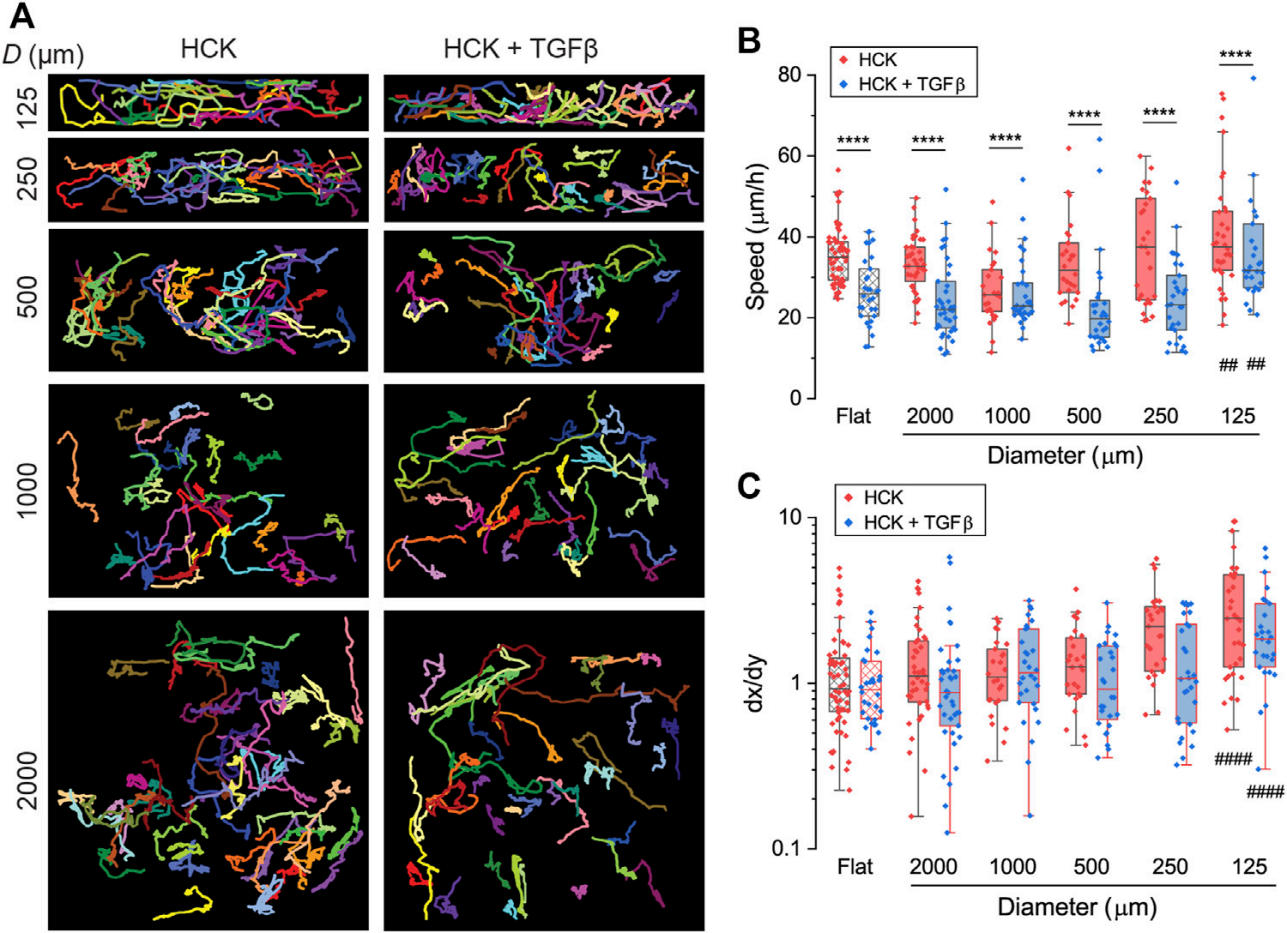
Migration of HCK and HCK + TGFβ cells on concave cylinders. **(A)** Migration tracks of cells on concave cylinders with diameters between 125 and 2000 µm. Every colored track represents an individual cell, with at least 26 cells per condition. **(B)** Migration speed of individual cells (dots) on concave cylinders. **(C)** Directionality index dx/dy of individual cells (dots) on concave cylinders. ****: *p* < 0.0001 for comparisons between HCK and HCK + TGFβ cells for the same curvature; ^####^: *p* < 0.0001 for comparisons against flat control for either HCK or HCK + TGFβ cells; all other comparisons are not statistically significant.

To elucidate the effect of corneal keratocyte transdifferentiation on curvature-mediated cell migration, we tracked the migration of HCK with fibroblastic and myofibroblastic phenotypes (HCK and HCK + TGFβ) on concave cylindrical structures of a range of diameters (*D* = 125, 250, 500, 1000, and 2000 µm) with time-lapse confocal imaging. Migration tracks for each cell were then constructed (Figure 5A) and analyzed to quantify the cell migration characteristics. HCK cells were generally motile throughout the 22 h of migration tracking, with average speeds ranging from 10 to 80 μm/h. For all curvatures, including on flat surfaces, the HCK cell speed in the presence of TGFβ is significantly lower than that in the absence of TGFβ (Figure 5B, *p* < 0.0001). The cylinder curvature did not significantly affect cell speed, except for the smallest tested cylinder (*D* = 125 µm), which led to higher cell speed compared to on flat surfaces, both with and without TGFβ (*p* < 0.01).

We quantified the directionality of the migration using the directionality index dx/dy , which denotes the ratio between the maximum cell displacement in the longitudinal ( x ) versus circumferential ( y ) directions of the cylinder. On flat substrates, the cells had an average directionality index close to 1 as expected, indicating randomly oriented cell migration (Figure 5C, dx/dy = 1.26 ± 0.13 for HCK cells and 1.06 ± 0.10 for HCK + TGFβ cells). For both HCK cells with and without TGFβ, there was a general trend of increasing dx/dy with decreasing cylinder size, albeit without statistically significant difference (*p* > 0.05). The directionality index only became significantly larger than that on the control flat surface on cylinders with a diameter of 125 µm (Figure 5C, dx/dy = 3.45 ± 0.55 for HCK cells and 2.23 ± 0.28 for HCK + TGFβ cells, *p* < 0.0001). This indicates that, with increasing substrate curvature, the cells increasingly preferred to migrate along the longitudinal cylinder axis. No statistically significant difference was found between HCK cells with and without TGFβ treatment on the same cylinder curvatures.

**FIGURE 6:**
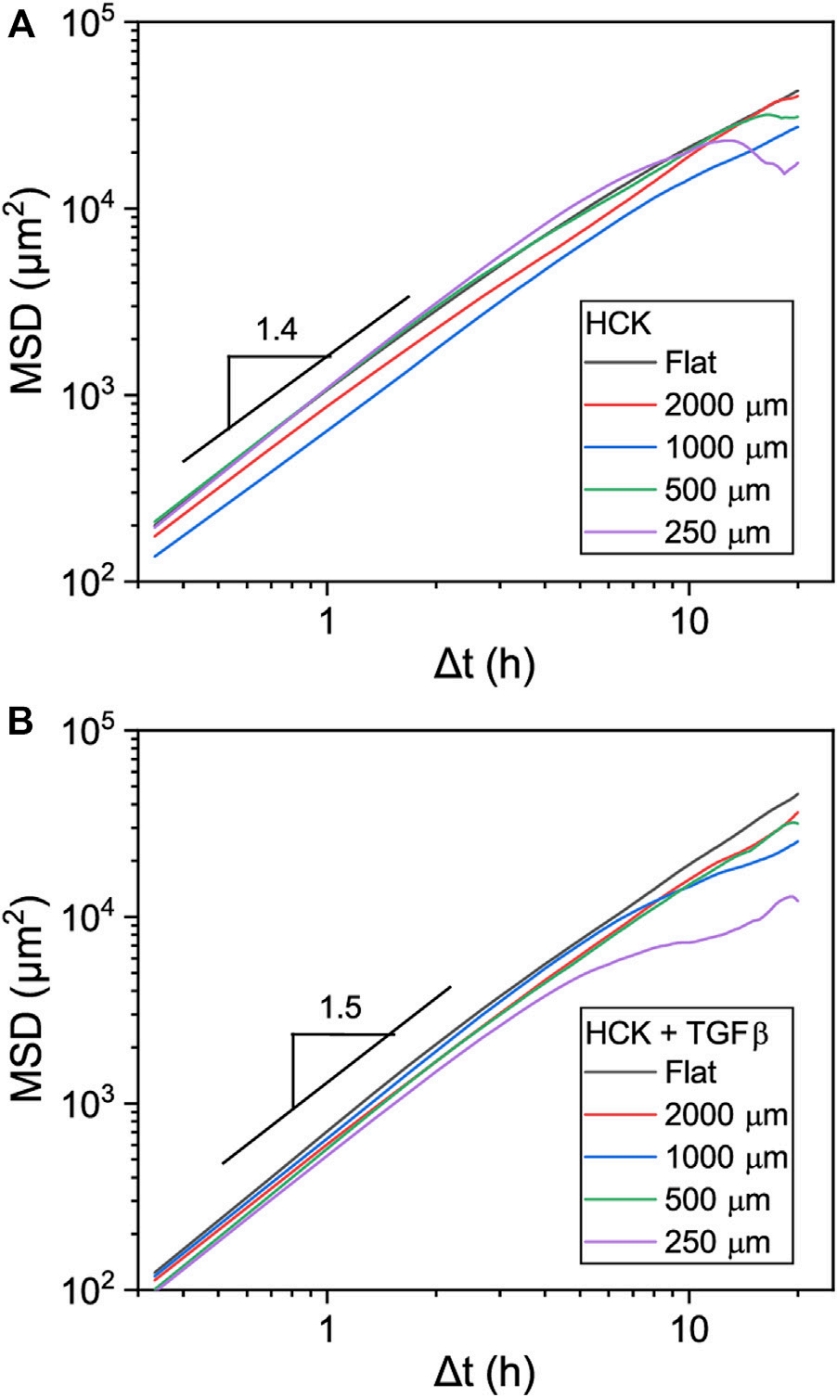
Mean squared displacement (MSD) of **(A)** HCK and **(B)** HCK + TGFβ cells, migrating on cylindrical structures of the indicated sizes. Logarithmic slopes of 1.4 and 1.5 are shown as an indication. Data for *D* = 125 µm was not analyzed due to the relatively high noise level in the MSD.

To gain further insight into the migration phenotype of the HCK cells, we calculated the mean-squared displacement (MSD) of the migration tracks. At short time intervals ( Δt < 5 h), the MSD followed $\Delta t^{\alpha }$ with logarithmic exponent α > 1 (Figure 6). This superdiffusive behavior at short time intervals is consistent with model predictions for directionally persistent motion of cells (Loosley et al., 2015). The exponent gradually decreased towards longer time intervals, as expected (Dieterich et al., 2008). TGFβ appeared to slightly affect the exponent α , with α ∼ 1.4 for HCK cells and α ∼ 1.5 for HCK + TGFβ cells, but the overall time dependence did not appear to vary between different curvatures, suggesting that varying substrate curvatures did not induce qualitatively different migration modes.

### Human corneal keratocyte cell migration directionality is affected by substrate curvature, but not in the presence of TGFβ

**FIGURE 7:**
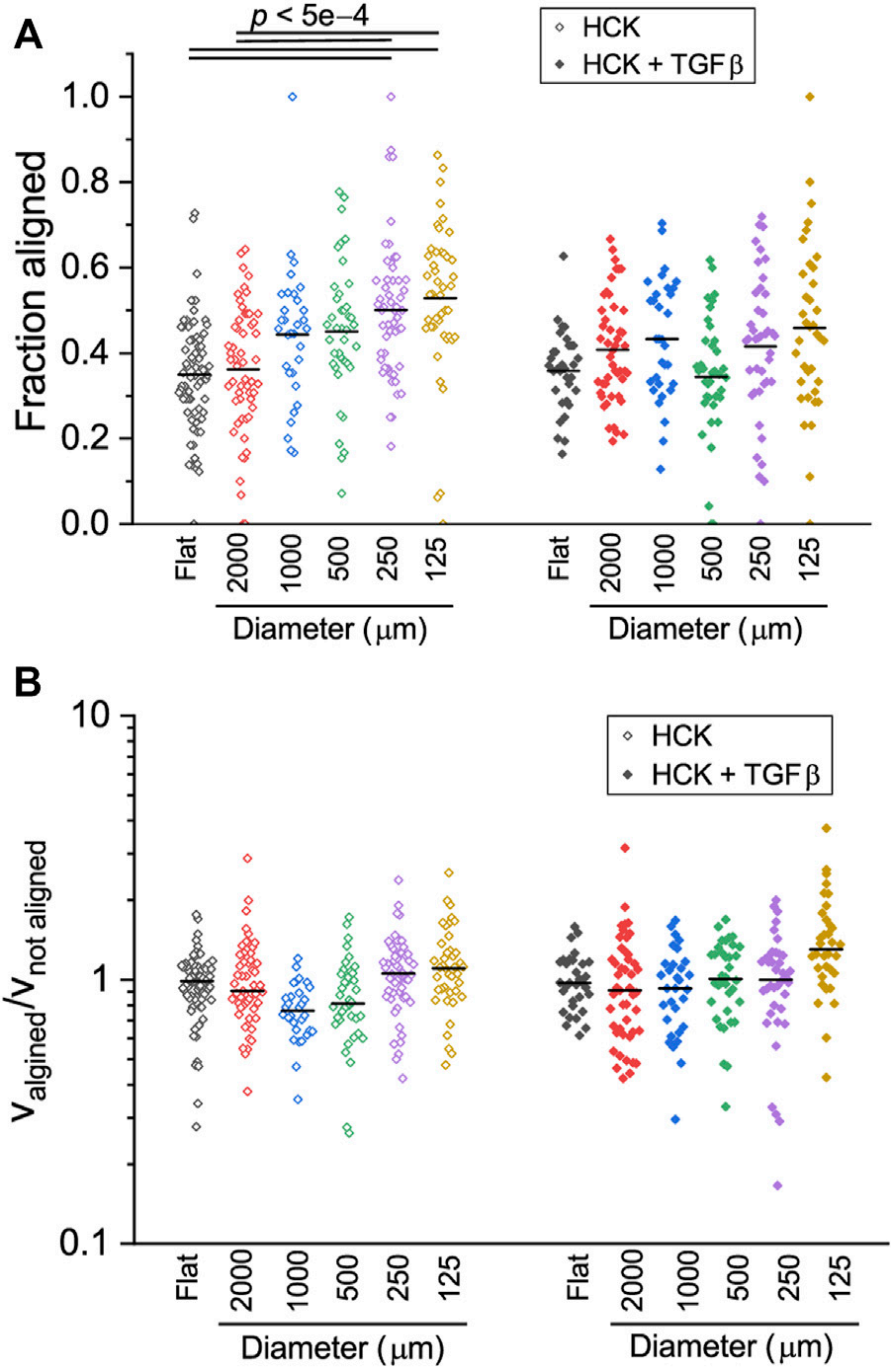
Direction-dependent migration of HCK and HCK + TGFβ cells on concave cylinders. **(A)** Fraction of time that cells migrated along the longitudinal axis of the cylinder. HCK cells appear to spend more time on aligned migration with increasing curvature. **(B)** Ratio of cell speed when aligned or non-aligned, indicating the no differences in speed for HCK and HCK + TGFβ cells on all concave cylinders. Each data point represent one tracked cell (*n* > 30 for each condition). Statistically significant differences are indicated with the *p* values; all other paired comparisons did not show statistically significant difference.

The uniqueness of cylindrical structures is the direction-dependent curvature, which allows cells to reorient their cell bodies to adjust to the mechanical demand associated with the non-planar substrate geometry. Previous works have shown that cells orient their cell bodies either in the longitudinal or circumferential directions of cylindrical substrates depending on the cell type, sign and magnitude of the curvature (Fioretta et al., 2014; Liu et al., 2018; Yip, Huang and Chiam, 2018; Werner et al., 2019), and this translates to migration direction (Werner et al., 2019). We therefore hypothesized that the curvature effects on HCK cells can be better understood by examining in detail the direction-dependent trajectories of the cells. First, we quantified the amount of time that the cells spent migrating in the longitudinal direction of the cylinders, relative to the total migrating time. We found that this fraction steadily increases with increasing curvature (i.e., decreasing cylinder size, *p* < 0.0005) for HCK cells, reaching 0.53 ± 0.03 on 125 µm (Figure 7A). In contrast, no statistically significant change was found between different curvatures for HCK cells in the presence of TGFβ (*p* > 0.05).

**FIGURE 8:**
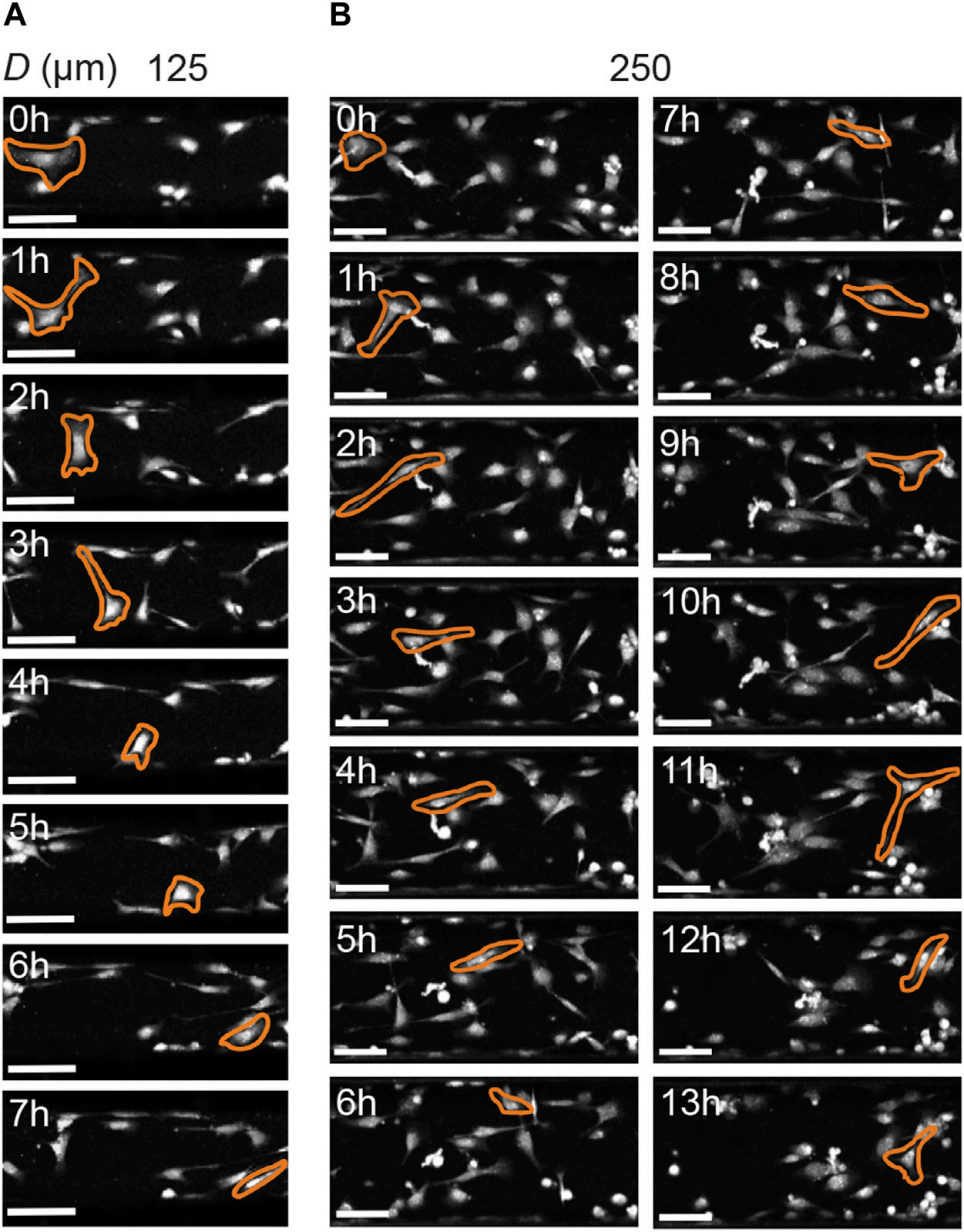
Cell morphology of HCK cells on concave cylinders (longitudinal axis from left to right) during migration. **(A)** Cells actively probe all directions on the smallest cylinder (125 µm), yet still favor migration in the longitudinal direction of the cylinder. **(B)** Similar observations for HCK cells on a larger cylinder (250 µm), where cells actively form protrusions in the circumferential direction, but favor migration along the cylinder axis. Scale bars: 100 µm.

Previous work on human mesenchymal stromal cells demonstrated that cells can dynamically adjust their migrating speed depending on their instantaneous orientation (and therefore perceived curvature), with mesenchymal stromal cells migrating faster when oriented in higher negative (i.e., concave) curvature (Werner et al., 2019). To test whether this is also the case for HCK cells, we quantified the ratio between cell speed when aligned versus cell speed when not aligned. Interestingly, this ratio remained close to one for all curvatures, both with and without TGFβ, with no statistically significant difference across different conditions (Figure 7B, *p* > 0.05). A closer inspection of the cell morphology dynamics during migration on the two smallest cylinders (125 and 250 µm) indicate that, while cells dynamically form protrusions to probe the curved substrate in the circumferential direction, they often retract these protrusions in favor of migrating in the longitudinal direction, where the perceived curvature is zero (Figure 8). Together, these results suggest that HCK cells do not change their migration mode and speed on different magnitudes of concave substrates, but actively reorient to adjust to the substrate curvature, and this curvature-dependent migration behavior is affected by myofibroblastic transdifferentiation.

## Discussion

Approximately 1.9 million patients worldwide are blind due to corneal opacity, and tens of millions have partially impaired vision (Burton, 2009). Currently available treatments include corneal transplantation, phototherapeutic surgery, and anti-inflammatory drugs, but are still far from ideal. Corneal transplants risk immunogenic response and limited availability of donor tissue, surgical procedures regularly cause collateral damage to the eye, and drugs often remain ineffective (Chaurasia et al., 2015). There is a need for a fundamental understanding of the behavior of human corneal keratocytes as the main cellular player in the healing and regeneration of the cornea, in order to develop treatments that can fully restore the structure and functions of the corneal stroma without causing fibrosis. The effect of myofibroblastic transdifferentiation in the keratocyte response to geometrical curvature cues relevant to the cornea has not been investigated before, and formed the focus of our present study.

Myofibroblastic transdifferentiation is often induced in *in vitro* culture through TGFβ treatment, leading to hallmarks of myofibroblast phenotype such as extensive stress fibers, mature focal adhesions, expression of α-SMA, and increased contractile forces (Tomasek et al., 2002; Zent and Guo, 2018; Vallée and Lecarpentier, 2019). Here we used the HCK cell line in combination with TGFβ supplementation to have corneal fibroblastic and myofibroblastic cell culture models. The experimental protocols have been optimized and validated in a previous study for the HCK cell line (Manzer et al., 2009) and is similar to those used in another study with primary keratocytes (Kim et al., 2010). Curvature was imposed using 2.5D chips containing cylindrical structures of varying dimensions, coated with fibrillar collagen to mimic corneal stromal tissue architecture. The geometry of curved substrates provides cells with non-planar, 3D-like structural cues not present in conventional 2D cell-culture systems, which are known result in differences in cell morphology and attachment (Friedl and Bröcker, 2000; Baker and Chen, 2012).

We found that fibroblastic and myofibroblastic corneal keratocytes had trouble adhering to convex substrates, although they adhered normally on flat and concave substrates. It has been proposed using a mechanical model that cell orientation on curved substrates is mediated by a trade-off between stress fiber bending energy and deformation energy in the cell as a whole due to active contractility (Biton and Safran, 2009). Cells with developed stress fibers, such as active fibroblasts, try to minimize the stress fiber bending energy by avoiding bending of the cell body and compression of the nucleus enforced by substrate curvature. This can be done for example by migrating away from convex areas towards concave areas (Pieuchot et al., 2018) or by reorienting in the direction of least convex curvature (Werner et al., 2019). The minimization of stress fiber bending could be the reason for the failure of HCK cells to adhere on convex substrates. Indeed, when the culture was maintained for a prolonged time, high cell density in other areas forced the cells to migrate onto the convex areas, though the cells did not show healthy morphology.

On concave substrates, both fibroblastic and myofibroblastic corneal keratocytes were adhering and actively migrating. We observed a general trend of higher migrating speed and directionality along the longitudinal axis with higher concave curvatures, especially for the highest tested curvatures (*D* = 125 µm). This demonstrates that both cell types were able to sense and respond to geometrical curvature cues in the environment. Yet, the concave curvature dependence of cell speed and MSD for fibroblastic and myofibroblastic corneal keratocytes was relatively weak compared to the difference between the two cell types. This highlights the importance of cell phenotype in governing attachment and migration behavior. Fibroblastic keratocytes exhibit strong, assembled stress fibers that can explain the curvature avoiding behavior. In comparison, the cellular contractility and attachment strength of the more activated myofibroblastic keratocytes are likely to be increased upon stimulation of the Rho pathway by TGFβ (Goffin et al., 2006; Kim et al., 2010), resulting in attenuated curvature avoidance response. Consistent with this, we found that, for the fibroblastic keratocytes, the fraction of time spent migrating in an aligned fashion was affected much more strongly by curvature than that for the myofibroblastic keratocytes.

Taken together, our study demonstrates that myofibroblastic transdifferentiation can strongly affect the adhesion and migration phenotypes of corneal keratocytes as well as their sensing of and response to geometrical cues in the tissue environment. In particular, myofibroblastic keratocytes have stronger substrate adhesion, switch to a slower migration, and have weaker curvature sensing. This is relevant not only in terms of the fundamental understanding of keratocyte behavior during wound healing or fibrosis of the corneal stroma, but also in terms of potential biomedical applications. Tissue engineering of the corneal stroma has emerged as a promising strategy for treating corneal diseases (Ghezzi, Rnjak-Kovacina and Kaplan, 2014; Matthyssen et al., 2018). In this approach, biomaterial scaffolds can be rationally designed and tuned to instruct cells to regenerate their own ECM (Kurniawan, Chaudhuri and Lim, 2016) and prevent keratocyte transdifferentiation into myofibroblasts, using microscale topographical cues such as microgrooves (Xiong et al., 2019) or macroscale geometrical cues such as those investigated in the present study. The range of curvatures used in this study (1/62.5 ≥ κ ≥ 1/1000 μm^−1^) was chosen to allow comparison to previous studies on the effect of curvature on cell behaviors (Werner et al., 2018; 2019; van der Putten et al., 2021), but was higher than that in the cornea of human adults ( κ = 1/8000 μm^−1^). It will be interesting to examine the keratocyte response on curvatures that more closely match those of healthy and diseased human adult corneas. Furthermore, by controlling the orientation and organization of the corneal keratocytes, the structure of the natural corneal stroma with regularly aligned collagen lamellae on the microscale as well as the overall curved shape may be replicated (Gouveia et al., 2017). It is anticipated that a combination of geometrical cues at different scales can provide a powerful tool to guide cell behavior towards healthy wound healing and tissue regeneration. We finally note that, although the HCK cell line used in this study have been used as a faithful human cornea model, for instance in drug absorption and tissue engineering studies (Engelke et al., 2013; Grobe and Reichl, 2013; Kölln and Reichl, 2016; Türker et al., 2018), future clinical translations of our findings would require verification with primary cells and further animal studies.

## Data Availability

The raw data supporting the conclusions of this article will be made available by the authors, without undue reservation.
