# Supplementary material for: Myofibroblast transdifferentiation of keratocytes results in slower migration and lower sensitivity to mesoscale curvatures
Source: Front Cell Dev Biol. 2022 Jul 22;10:930373. doi: 10.3389/fcell.2022.930373 (PMC9355510; doi:10.3389/fcell.2022.930373)
Supplement: Supplementary file 1 [file DataSheet1.DOCX]

Supplementary Material

Myofibroblast transdifferentiation of keratocytes results in slower migration and lower sensitivity to mesoscale curvatures

Cas van der Putten^1,2†^, Daniëlle van den Broek^1,2†^, Nicholas A. Kurniawan^1,2*^

^1^Department of Biomedical Engineering, Eindhoven University of Technology, Eindhoven, the Netherlands

^2^Institute for Complex Molecular Systems, Eindhoven University of Technology, Eindhoven, the Netherlands

^†^These authors have contributed equally to this work and share first authorship

*** Correspondence:**Nicholas A. Kurniawan
n.a.kurniawan@tue.nl

# Supplementary Figures


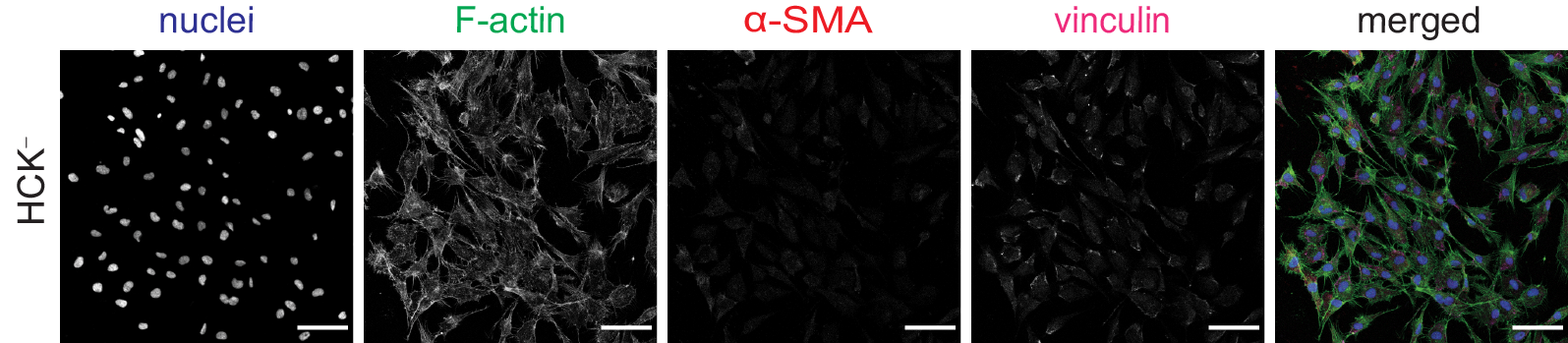


**SUPPLEMENTARY FIGURE 1 |** Immunofluorescent staining of HCK^−^ cells, showing a non-activated phenotype. Cells are stained for nuclei (Dapi, blue), F-actin (phalloidin, green), α-SMA (green), and vinculin (magenta). Scale bar: 100 µm.


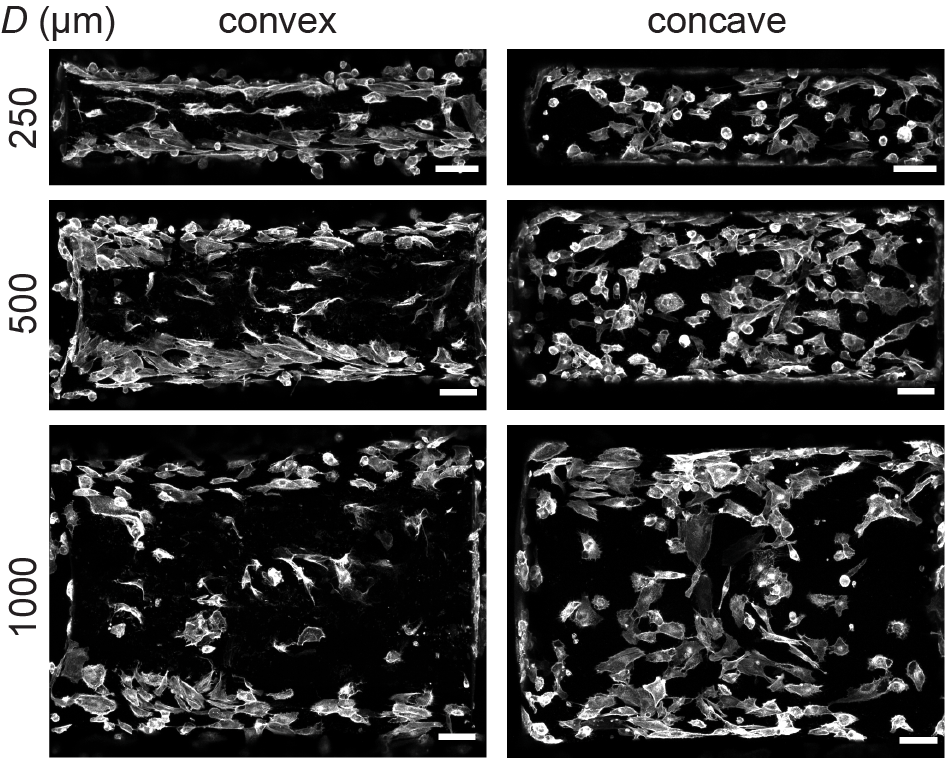


**SUPPLEMENTARY FIGURE 2 |** HCK cell adhesion on convex and concave cylinders. Cells were stained for F-actin after 4 days of culture. Although HCK cells eventually adhere to the convex cylinders, almost all cells show an unhealthy morphology. Scale bars: 100 µm.
